# Ebola, Through Air or Not Through Air: That is the Question

**DOI:** 10.3389/fpubh.2014.00292

**Published:** 2015-01-19

**Authors:** Samuel Ponce De Leon-Rosales, Rene Arredondo-Hernandez, Alejandro Macias, Richard P. Wenzel

**Affiliations:** ^1^Division de Investigación, Facultad de Medicina, Universidad Nacional Autónoma de México, Mexico City, Mexico; ^2^Departamento de Medicina y Nutrición, Universidad de Guanajuato, Guanajuato, Mexico; ^3^Division of Infectious Diseases, Department of Internal Medicine, Virginia Commonwealth University, Richmond, VA, USA

**Keywords:** Ebola virus, border line transmission, aerosolized potential, safety measures, variable contingency

The recent cases of Ebola contagion in health care workers make evident the lack of a complete understanding of the infection process. There are evidences against respiratory system involvement along the complete transmission cycle, from an infected individual to an uninfected one by the airborne/droplet transmission route.

Main stream knowledge have been the basis for actions and opinions by agencies such as the CDC, however, accelerated response may have overlooked gray areas and missed possible risk scenarios. Among the most challenging ones, and which could gain relevance as contact precautions are set is the accidental aerosol/droplet transmission.

Data supporting this claim sustains on experimental transmission with animal models, in addition to pathology and environmental microbiology findings.

Explanatory analysis accounts mostly to the susceptibility of exposed respiratory and mucous membranes to a source of low dose aerosolized Ebola virus to infect by inhalation or autoinoculation via water or fomites in non-human primates even when direct contact to the source is prevented (1, 2).

Even more, Ebola major symptoms, exception made for respiratory localized lesions, are quite similar no matter peritoneal or aerosol respiratory experimental inoculation in non-human primates (3). In humans, suspicion of aerosol transmission is reinforced because of lung-infected macrophages in post-mortem fixed tissue (4).

A fact, connecting both susceptibility and indirect transmission are that the Zaire Ebola virus decay rate in a dynamic aerosol would have allowed transmission. As an example, starting with almost 1000 infectious Zaire Ebola virus, after 90 min, enough viruses remain infective to cause disease. Moreover, if adsorbed dry on glass or plastic at low temperature, number of infective particles that would remain infective could be as high as up to 1 × 10^3^ times the reported minimal infectious dose for days (5).

Speculatively, air suspension or un-apparent surface deposit could plausibly explain epidemiologic research in which exposure source is not found for a number of patients (6).

Although current epidemic curves and spread rate at West Africa are consistent with contact, person-to-person transmission, rather than an epidemic pattern similar to that of diseases that have airborne/droplet transmission such as influenza or SARS, an alternative mode of transmission may be possible in particular situations.

In healthcare facilities, our infection control precautions need to be very schematic according to our understanding of suspected transmission mechanisms such as, airborne, droplet, or contact (7), but flexible enough to cope with conditional emerging risks. Being so, precautions are presented as one or the other (standard, droplet, airborne, or contact precautions), (1) airborne particles come in two sizes – those 10 μm in size or less that stay in the air and those over 10 μm in size that fall after about 6 feet; either could occur and be infectious in late stages of diseases. The infections that occurred in health care workers from Madrid and Dallas should be analyzed not only in regard of faults in the compliance with the precautions but also in the directions given in extreme contact precautions (8).

The Ebola-infected patient reaches at some point a high viral load in all body liquids and secretions due to several immunoevasion mechanisms resulting in unrestricted viral replication and dissemination (9). (2) If we consider that the infectious dose may be very low – at only 10 virus particles (3) – and that an airborne particle may contain thousands of Ebola virions, thus an exposure carried by air can be risky. Due to symptoms such as vomiting, coughing, bleeding, and defecating, not to mention defective respirator pumping devices, or even syringe plunges, splashes, and aerosols are easily created (10). If the virus requires an extremely low inoculum to cause infection and some viral particles can persist infective in the airborne droplet nuclei, a measurable risk of airborne transmission would be present, even keeping compliance with contact precautions.

At the time, this note was submitted, an ending of Ebola virus epidemic in West Africa is still uncertain, even so, attending the lessons of environmental epidemiology, distinguishing communalities and differences in the context transmission take place is on sake of identifying strategies to cut off efficient and/or un-efficient means of dispersion. Therefore, we should separate two different settings. One is the African transmission in which contact is the most probable cause of the explosive contagion. The other implies the transmission in spite of the contact precautions in place because of patients with high viral load generating aerosols or droplets.

Certainly, this hypothesis should be tested, but for time being the repeated transmission in the described setting warrants considering airborne/droplet transmission at some moment of the disease. The precaution emphasis may be widened and negative pressure rooms may be implemented for these patients.

## Conflict of Interest Statement

The authors declare that the research was conducted in the absence of any commercial or financial relationships that could be construed as a potential conflict of interest.
